# Should obesity be associated with worse urinary continence outcomes after robotic-assisted radical prostatectomy? a propensity score matching analysis

**DOI:** 10.1590/S1677-5538.IBJU.2021.0457

**Published:** 2021-08-10

**Authors:** Thiago Camelo Mourão, Renato Almeida Rosa de Oliveira, Ricardo de Lima Favaretto, Thiago Borges Marques Santana, Carlos Alberto Ricetto Sacomani, Wilson Bachega, Gustavo Cardoso Guimarães, Stênio de Cássio Zequi

**Affiliations:** 1 Hospital da Beneficência Portuguesa de São Paulo Departamento de Uro-Oncologia São Paulo SP Brasil Departamento de Uro-Oncologia, Hospital da Beneficência Portuguesa de São Paulo, São Paulo, SP, Brasil; 2 AC Camargo Cancer Center Fundação Antônio Prudente Escola de Pós-Graduação São Paulo SP Brasil Escola de Pós-Graduação, Fundação Antônio Prudente, AC Camargo Cancer Center, São Paulo, SP, Brasil; 3 AC Camargo Cancer Center Divisão de Urologia São Paulo SP Brasil Divisão de Urologia, AC Camargo Cancer Center, São Paulo SP, Brasil; 4 Hospital da Beneficência Portuguesa Departamento de Oncologia Cirúrgica São Paulo SP Brasil Departamento de Oncologia Cirúrgica, Hospital da Beneficência Portuguesa de São Paulo, SP, Brasil

**Keywords:** Obesity, Urinary Tract, Robotic Surgical Procedures

## Abstract

**Purpose:**

To analyze the association between obesity and urinary incontinence rate in men submitted to robot-assisted radical prostatectomy (RARP) in a high-volume cancer center.

**Materials and Methods:**

We reported 1.077 men who underwent RARP as the primary treatment for localized prostate cancer from 2013 to 2017. Patients were classified as non-obese (normal BMI or overweight) or obese men (BMI ≥30kg/m^2^). They were grouped according to the age, PSA level, D’Amico risk group, Gleason score, ASA classification, pathological stage, prostate volume, salvage/adjuvant radiotherapy, perioperative complications, and follow-up time. Urinary continence was defined as the use of no pads. For the analysis of long-term urinary continence recovery, we conducted a 1:1 propensity-score matching to control confounders.

**Results:**

Among the obese patients, mean BMI was 32.8kg/m^2^, ranging 30 - 45.7kg/m^2^. Only 2% was morbidly obese. Obese presented more comorbidities and larger prostates. Median follow-up time was 15 months for the obese. Complications classified as Clavien ≥3 were reported in 5.6% of the obese and in 4.4% of the non-obese men (p=0.423). Median time for continence recovery was 4 months in both groups. In this analysis, HR was 0.989 for urinary continence recovery in obese (95%CI=0.789 - 1.240; p=0.927).

**Conclusions:**

Obese can safely undergo RARP with similar continence outcomes comparing to the non-obese men when performed by surgeons with a standardized operative technique. Future studies should perform a subgroup analysis regarding the association of obesity with other comorbidities, intending to optimize patient counseling.

## INTRODUCTION

According to the World Health Organization (WHO), there were 1.9 billion overweight adults in the World in 2016, rising almost threefold since the 70s. In Brazil, the WHO estimated an increased age-standardized rate of obesity (body mass index [BMI] ≥30kg/m^2^) from 5.2% in 1975 to 22.1% in 2016. This high prevalence is translated into a common situation among urological patients.

In the literature, some studies have suggested an association between obesity and high-grade prostate cancer (PCa) (1, 2). The REDUCE study showed an increased risk of 28% in a multivariable analysis associated with high-grade PCa and a lower risk associated with low-grade PCa (3).

Obese patients treated with radical prostatectomy have conflicting results related to functional outcomes. According to some studies, the rates of postoperative urinary incontinence (UI) vary among different techniques (open, laparoscopic, or robot-assisted) (4–8). After the spread of robot-assisted radical prostatectomy (RARP), urological surgeons seem to be more confident about the functional outcomes of obese patients.

This study aimed to analyze a cohort of patients who underwent RARP at a high-volume cancer center and compare the rates of the postoperative recovery of urinary continence between obese men and a propensity score-matched group of non-obese patients.

## MATERIALS AND METHODS

Patient selection and study design

**Figure 1:**
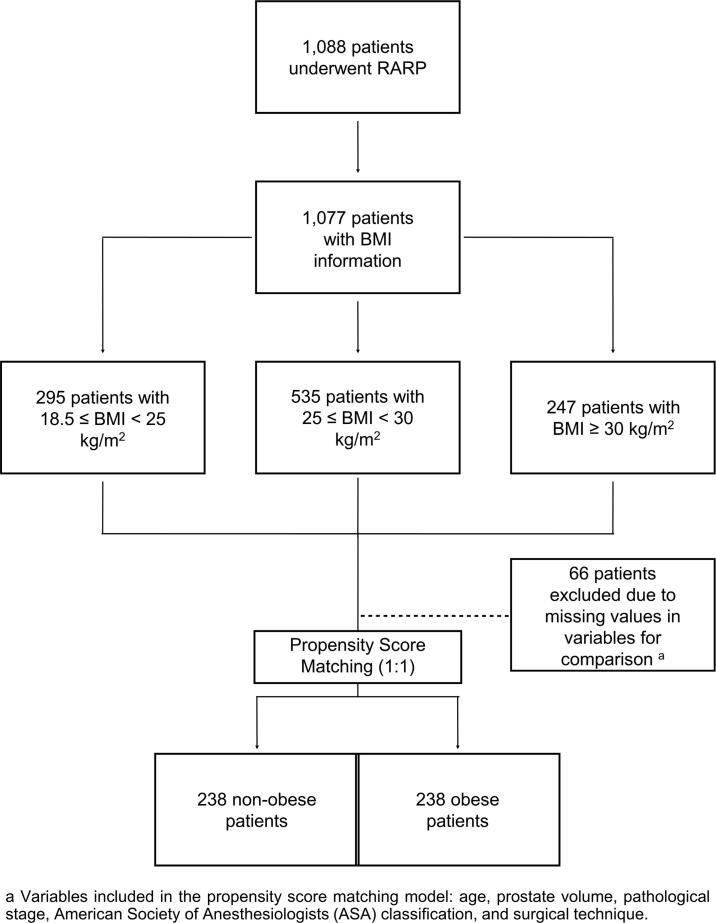
Flowchart of the study.

We collected the data from 1.088 patients who underwent RARP as the primary treatment of PCa from May 2013 to December 2017 in a single reference cancer center. The authors collected the data from the preoperative and postoperative appointments described in the patient charts and their respective pathological reports. Figure-1 shows the flowchart of the study.

In this cohort, the procedures from 13 urological surgeons were included after the completion of their training. Between 2013 and 2014, the patients underwent a transperitoneal RARP technique, including the incision of the endopelvic fascia and the dorsal venous complex control with hemostatic suture. Thereafter, all the patients underwent an extraperitoneal RARP procedure with the complete preservation of the anterior periprostatic tissue (9).

The patients were diagnosed with PCa by transrectal ultrasound-guided prostate biopsy at a minimum of six weeks before the surgery. For oncological staging, multiparametric magnetic resonance imaging (mpMRI) was performed in all patients. Furthermore, bone or PSMA-PET/CT scans were performed at the discretion of each surgeon in selected patients. Metastatic patients were excluded from this analysis.

The demographic, clinical, and pathological data were collected to compare the groups according to the BMI classification. Normal BMI was determined in patients with a value greater than or equal to 18.5 to 24.9kg/m^2^. Patients with a BMI greater than or equal to 25 to 29.9kg/m^2^ were regarded as overweight, while patients with a BMI greater than or equal to 30kg/m^2^ were considered as obese. Other grouped data were age, serum PSA level, D’Amico risk group, Gleason score, American Society of Anesthesiologists (ASA) risk classification, pathological tumor stage (pT), and follow-up time. Prostate volume was measured by the mpMRI, and salvage or adjuvant radiotherapy was indicated for locoregional or biochemical recurrences.

Routinely, the urinary catheter was removed seven days after the surgery, and the postoperative follow-up was conducted within 15 days, for one month, and then quarterly. Urinary continence was defined as the use of no pads, and continence status was determined through direct patient interviews during the postoperative appointments. The patients who complained of UI were referred to a pelvic floor rehabilitation after the first postoperative month. All the perioperative complications were graded according to the modified Clavien-Dindo classification. The study obtained Institutional Review Board approval.

### Statistical analysis

The data were summarized with medians and interquartile ranges (IQR) for nonparametric continuous variables. The categorical variables were presented as absolute numbers and percentages. Moreover, the Kruskal-Wallis test was used for the analysis of the nonparametric variables. The Fisher exact test or the Pearson chi-squared test was also employed for categorical variables when appropriate. A 1:1 propensity score matching (PSM) approach was utilized to control the confounders for the analysis of the urinary continence recovery. In addition, PSM was calculated by logistic regression. The nearest-neighbor matching model was based on age, prostate volume, pT stage, ASA risk classification, and surgical technique. The missing values of these variables were excluded before the analysis (Figure-1). The Kaplan-Meier method and the Cox proportional hazard model were used for the analysis, and the groups were compared by employing the log-rank test. Additionally, we considered the time zero as the date of the surgery, and the significance level was established for P-value <0.05. Statistical analysis was performed using IBM SPSS Statistics version 23.0 (IBM Corp., Armonk, NY) and R software version 4.0.0 (10). A supplementary table was included with data from the patients after the PSM method.

## RESULTS

Table-1 presents the baseline demographic, clinical, and pathological data for the 1.077 patients grouped by the BMI classification. Almost half of the cohort (49.7%) were overweight patients. Among the obese patients (247 patients), the mean BMI was 32.8kg/m^2^, ranging from 30.0 to 45.7kg/m^2^. The majority of the obese patients were classified as class I obese, and only 2% were considered morbidly obese (BMI ≥40kg/m^2^).

**Table 1:** Overall demographic, clinical, and pathological data according BMI classification.

N (Total = 1077)		Normal BMI	Overweight	Obese	P - value
		295 (27.4%)	535 (49.7%)	247 (22.9%)	
Median age (IQR)		62 (57 – 68)	61 (56 – 67)	61 (57 – 66)	0.173 a
PSA value (ng/mL)		5.4 (4.0 – 7.84) N=262	5.47 (4.01 – 7.75) N=484	5.53 (4.01 – 8.4) N=221	0.764 a
**Gleason score**					0.261 b
	≤ 6	34 (11.6%)	59 (11.1%)	24 (9.7%)	
	7	216 (74%)	376 (70.9%)	169 (68.4%)	
	8 - 10	42 (14.4%)	95 (17.9%)	54 (21.9%)	
**D’Amico Risk group**					0.277 b
	Low Risk	103 (38.6%)	164 (33.3%)	74 (33.9%)	
	Intermediate Risk	84 (31.5%)	176 (35.7%)	65 (29.8%)	
	High Risk	80 (30%)	153 (31%)	79 (36.2%)	
**ASA classification**					**< 0.001**^b^
	I	61 (20.7%)	81 (15.2%)	11 (4.5%)	
	II	213 (72.2%)	406 (76.3%)	204 (82.6%)	
	III	21 (7.1%)	45 (8.5%)	32 (13%)	
Prostate volume (cm^3^)		39 (30 – 48) N=277	41 (34 – 51) N=499	42 (34 – 54) N=238	**0.005**^a^
**Pathological stage**					0.327 b
	pT2	226 (77.7%)	397 (75%)	178 (72.1%)	
	pT3	65 (22.3%)	132 (25%)	69 (27.9%)	
Median F-U (months)		13 (6 – 27)	15 (6 – 27)	15 (6 – 28)	0.824 a
**Salvage or Adjuvant RT**					0.937 b
	Yes	24 (12.7%)	43 (12%)	21 (13.1%)	
	No	165 (87.3%)	314 (88%)	139 (86.9%)	

**BMI** – Body mass index; **PSA** – Prostate-specific antigen; **ASA** – American Society of Anesthesiologists; **F-U** – Follow-up; **RT** - Radiotherapy

a Kruskal-Wallis test

b χ^2^ test

Significant differences among the groups were evidenced for prostate volume and ASA classification. The obese patients presented with larger prostates. Prostates over 80cm3 were observed in 6.3% (15 cases) of the obese patients and in 2.7% (21 cases) of the non-obese patients. As expected, the obese patients presented with a higher frequency of concurrent comorbidities (patients classified as ASA II or ASA III). This is correlated with more comorbidities among obese patients and in more severe systemic diseases within this group.

In this cohort, the other analyzed variables did not display significant differences among patients with normal BMI, overweight patients, and obese patients. Related to the postoperative follow-up period, 57.6% (132 cases) and 35.8% (82 cases) of the obese patients had a minimum of 12 months and 24 months of follow-up, respectively. The non-obese patients presented at least 12 months and 24 months of follow-up in 58.6% (446) and 33.7% (257) of the cases, respectively.

Perioperative complications were also reported, and they were classified according to the Clavien-Dindo classification in Table-2. There were no significant differences between the obese and non-obese patients (p=0.423). Complications classified as Clavien ≥3 were reported in 5.6% of the obese patients and in 4.4% of the non-obese patients.

**Table 2:** Comparison of overall perioperative complications for obese (BMI ≥ 30 kg/m^2^) and non-obese men (BMI < 30 kg/m^2^).

Clavien-Dindo classification	Non-obese	Obese	P - value
I	42 (5.1%)	12 (4.9%)	0.423 a
II	42 (5.1%)	20 (8.1%)	
IIIA	13 (1.6%)	8 (3.2%)	
IIIB	11 (1.3%)	3 (1.2%)	
IVA	10 (1.2%)	3 (1.2%)	
IVB	1 (0.1%)	0	
V	2 (0.2%)	0	

**BMI** – Body mass index

a Two-sided Fisher's exact test

**Figure 2:**
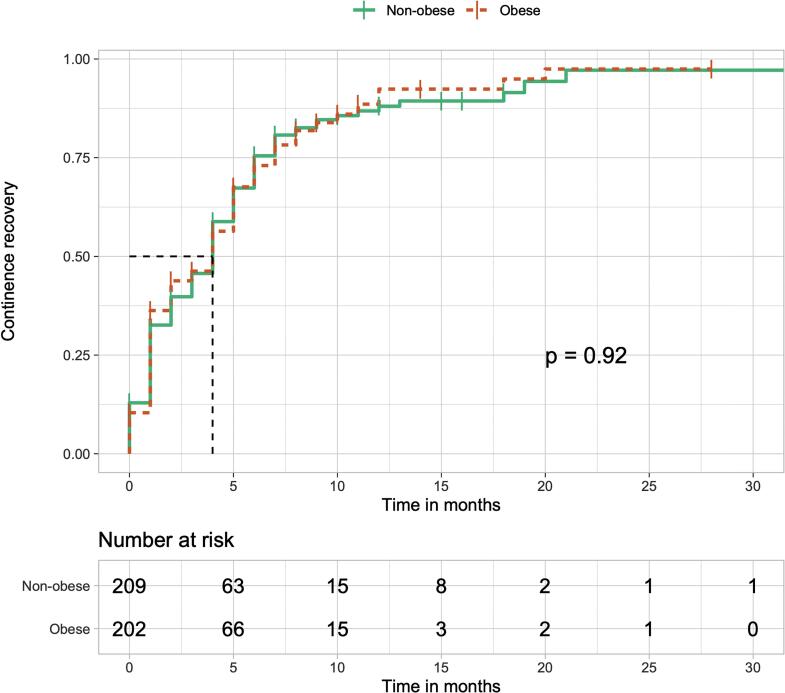
Kaplan-Meier curve of cumulative continence recovery rate between obese and non-obese patients.

The patients were categorized into two groups, namely, non-obese patients and obese patients, after the matching method. Non-obese patients included all cases with calculated BMI <30kg/m^2^. Figure-2 illustrates the cumulative urinary continence recovery between both groups. The median time for continence recovery was 4 months in both groups. In this cohort, there were no significant differences during the follow-up time (p=0.92). The Cox proportional hazard model was also assessed, and in this analysis, HR was 0.989 for urinary continence recovery in obese men (95% CI=0.789-1.240; p=0.927). Finally, it was conducted an analysis among the whole group of obese patients before the PSM approach. Obese men with localized disease presented a mean time for continence recovery of 4.63 months (3.65 - 5.61 months). In obese men with locally advanced disease, the mean time was 7.11 months (4.84 - 9.38 months). Regarding this comparison, it was evidenced a significant difference (p=0.040).

## DISCUSSION

Concerns associated with functional outcomes are still prevalent in the robotic setting. Frequently, surgeons overcoming their learning curve or in high-volume centers face patients with potentially adverse risk factors and poor functional or oncological outcomes. Even with the lack of high-level evidence, previous randomized trials are clear to show better perioperative outcomes and have provided support to confirm at least the comparable functional outcomes of RARP compared with laparoscopic or open approaches (11, 12). The overall prevalence of UI after RARP has been reported to be ranging from 4% to 31% (13), while the 12-month UI rate in obese has been reported to be ranging from 6% to 47% (8, 14–16).

Over the years, obese men have demonstrated a higher chance to undergo radiotherapy or brachytherapy, as a primary treatment, rather than radical prostatectomy. This has been evidenced by a study with patients from the CaPSURE database (17). Despite that, using a health-related quality-of-life questionnaire, an earlier study extracted from the same database has reported that there are worse urinary functions and bother among patients with a BMI of over 35kg/m^2^ (18).

A meta-analysis published in 2018 showed divergent results about postoperative UI. Two studies involving RARP and other two studies involving laparoscopic radical prostatectomy (LRP) were evaluated. The authors evidenced a significant association between obesity and UI at 12 months in the patients who underwent RARP (OR=2.43, 95% CI=1.21-4.88) and at 24 months (OR=2.00, 95% CI=1.57-2.56). However, the patients who underwent LRP did not show this association (4). Probably, this significantly better continence rate in non-obese men occurred due to the study of Wiltz et al. (15), which constituted 90% of the men who underwent RARP in the previously mentioned meta-analysis and exhibited similar conclusions.

In fact, considering studies prior to 2010, Boorjian et al. suggested that RARP offers similar functional outcomes for obese men (16). Other investigations have reported poorer postoperative continence rates or a longer time to continence recovery in obese men than in non-obese men (6, 14, 15). Analyzing more recent studies, we can come across with more authors affirming that there is no significant association between obesity and long-term UI rate (7, 8, 19). The study of Kumar et al. validated that the only significant divergence is about an earlier median time to continence recovery (19). Some possible explanations are that reference centers have achieved a higher expertise level over this period and that several technical improvements have been reported. Other discrepancies may occur due to the different techniques and continence definitions. A local study published in 2019 reported data from 104 consecutive RARP. In this series, only 16 patients were obese and there was no statistical difference in continence recovery compared to the non-obese men. However, the mean time to reach urinary continence was higher for the obese patients (5.08 months vs. 2.71 months) (20).

Considering morbid obesity, only a few studies have included this particular population, but none of them have reported functional outcomes over the follow-up period (21–23). In our study, it was evidenced a non-significant difference among class I to II obese and class III obese men (data not reported). Nonetheless, there are only 2% of morbidly obese patients in our cohort.

Several limitations can be discussed in this study. A debatable fact is that the group is composed of different surgeons in a single cancer center, who may have distinct surgical skills. Despite that, surgeons involved in this study overcame their learning curve with a standardized protocol and similar methods. They were included at the robotic program after assisting cases as bedside surgeons (24), completing the training on da Vinci system, and after approval of the head of the department. Some of them have a huge previous experience in open procedures. Others have been laparoscopists before the beginning of their learning curves with robotics. Additionally, findings are limited by the retrospective nature of the study and by the missing values in some cases, requiring exclusion for the PSM analysis. Despite that, we could control some confounders between both groups with matched analysis and compare them for an adequate follow-up period. Furtherly, we were not able to list the frequency of each one of the concurrent comorbidities in obese and non-obese men, which could be associated with worse UI status in particular scenarios. There are other potential conflictual covariates, such as race, educational level, or sedentary lifestyle, that could lead to divergent results. Another point is that only 2% of the cases consisted of morbidly obese, besides that super obese patients (BMI ≥50kg/m^2^) were not reported. Future studies with this specific population could lead to divergent outcomes. Finally, to our knowledge, this is largest national study analyzing the urinary functional outcomes in the population of obese men who underwent RARP.

## CONCLUSIONS

Obese patients can safely undergo robotic-assisted radical prostatectomy with similar continence outcomes compared with non-obese men. Our results suggest a comparable perioperative complication rate between the groups when performed by surgeons with a standardized operative technique. Future studies should perform a subgroup analysis regarding the association of obesity with other comorbidities, intending to optimize patient counseling.

## APPENDIX Supplementary Table – Data from obese and non-obese men included in the study after a propensity score matching approach.

**Table:**

ID case	Age	Weight	Height	BMI	Prostate volume	ISUP grade	PSA level	D′Amico classification	ASA classification	pT stage
1	62	103,0	1,820	31,10	59,0	2	9,25	Intermediate risk	2	pT2c
2	41	82,0	1,700	28,37	23,0	3	3,65	High risk	1	pT2c
3	58	92,0	1,720	31,10	47,0	1	6,78	Low risk	2	pT2c
4	54	95,0	1,720	32,11	40,0	1	5,54	Low risk	2	pT2c
5	65	82,0	1,600	32,03	42,0	3	8,15	High risk	2	pT2c
6	63	76,0	1,590	30,06	98,0	2	12,25	Intermediate risk	2	pT2c
7	63	120,0	1,750	39,18	37,0	1	5,78	Low risk	3	pT2c
8	56	102,0	1,900	28,25	50,0	1	11,00	High risk	3	pT2c
9	61	89,0	1,710	30,44	38,0	1	4,72	Low risk	2	pT2c
10	69	80,0	1,590	31,64	33,0	2	16,00	High risk	3	pT3a
11	75	93,0	1,730	31,07	60,0	4	5,20	High risk	2	pT3a
12	60	105,0	1,800	32,41	42,0	1	11,00	Low risk	2	pT2c
13	69	90,0	1,860	26,01	52,0	3	5,39	High risk	3	pT3b
14	60	100,0	1,720	33,80	49,0	4	20,70	High risk	2	pT3b
15	66	85,0	1,660	30,85	30,0	5	4,41	High risk	2	pT3b
16	61	120,0	1,620	45,72	49,0	N/A	N/A	N/A	2	pT2c
17	48	90,0	1,700	31,14	44,0	4	0,41	High risk	2	pT3a
18	73	78,0	1,780	24,62	48,0	2	1,20	Intermediate risk	3	pT3a
19	48	123,0	1,840	36,33	32,0	2	5,09	Intermediate risk	2	pT2c
20	72	96,0	1,790	29,96	50,0	N/A	N/A	N/A	3	pT2c
21	65	109,0	1,810	33,27	42,0	N/A	1,25	N/A	2	pT3b
22	68	91,0	1,760	29,38	44,0	5	62,00	High risk	3	pT2c
23	57	70,0	1,710	23,94	54,0	1	8,40	Low risk	2	pT2c
24	65	91,0	1,630	34,25	36,0	1	1,15	Low risk	2	pT3a
25	54	115,0	1,850	33,60	34,0	1	4,03	Low risk	2	pT2c
26	71	107,0	1,810	32,66	83,0	3	7,52	High risk	2	pT2c
27	58	105,0	1,850	30,68	24,0	1	3,02	Low risk	2	pT2c
28	68	73,0	1,730	24,39	72,0	1	11,90	Intermediate risk	2	pT2c
29	55	102,0	1,690	35,71	47,0	1	3,76	Low risk	3	pT2c
30	67	116,0	1,860	33,53	92,0	5	8,01	High risk	2	pT3b
31	61	98,0	1,790	30,59	54,0	5	3,36	High risk	2	pT3b
32	69	90,0	1,690	31,51	61,0	5	5,30	High risk	2	pT2c
33	62	120,0	1,760	38,74	31,0	5	7,90	High risk	2	pT3b
34	76	90,0	1,880	25,46	37,0	2	4,46	Intermediate risk	3	pT2c
35	55	81,0	1,740	26,75	41,0	1	5,49	Low risk	2	pT2c
36	69	111,0	1,800	34,26	52,0	1	2,68	Low risk	3	pT2c
37	61	94,0	1,640	34,95	54,0	2	6,50	Intermediate risk	2	pT2c
38	61	91,0	1,670	32,63	23,0	2	12,00	High risk	2	pT2c
39	72	90,0	1,670	32,27	61,0	2	18,00	High risk	3	pT3b
40	60	92,0	1,650	33,79	46,0	4	13,24	High risk	1	pT3b
41	72	93,0	1,720	31,44	31,0	2	6,96	Intermediate risk	2	pT2c
42	70	89,0	1,660	32,30	44,0	N/A	N/A	N/A	2	pT2a
